# Supplementary material for: The interfacial structure of water droplets in a hydrophobic liquid
Source: Nat Commun. 2017 May 24;8:15548. doi: 10.1038/ncomms15548 (PMC5458086; doi:10.1038/ncomms15548)
Supplement: Supplementary Information — Supplementary Figures, Supplementary Tables, Supplementary Note and Supplementary References [file ncomms15548-s1.pdf]

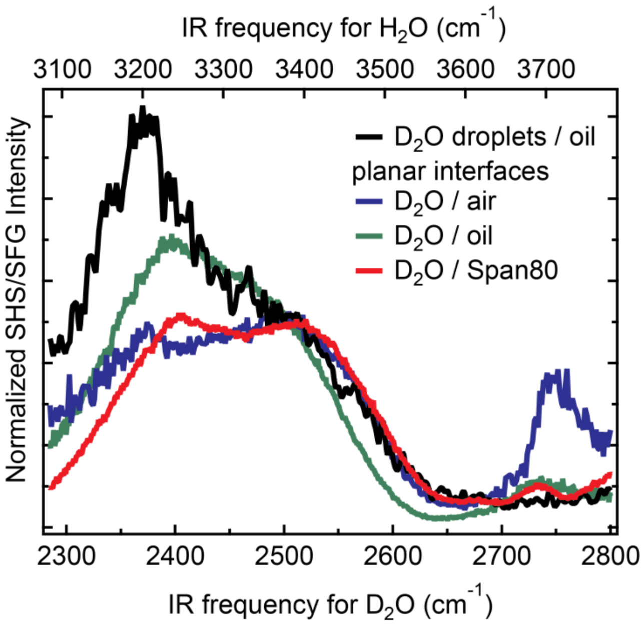

Supplementary Figure 1. Additional SFG data. Comparison of an SFS spectrum of water droplets in a hydrophobic liquid (black line, 1%v. D_2O in 5 mM Span80 in d_{34}-hexadecane) with SFG reflection spectra from D_2O/air (blue), D_2O/hexane (green) and D_2O/Span80 (red) interface. The SFG spectrum of the Span80/water interface is similar to that of D_2O/air, apart from the free OD mode that is significantly less intense. This indicates that Span80 cannot give rise to the enhanced strongly H-bonded peak and complete elimination of the free OD mode in SFS spectra of nanodroplets.

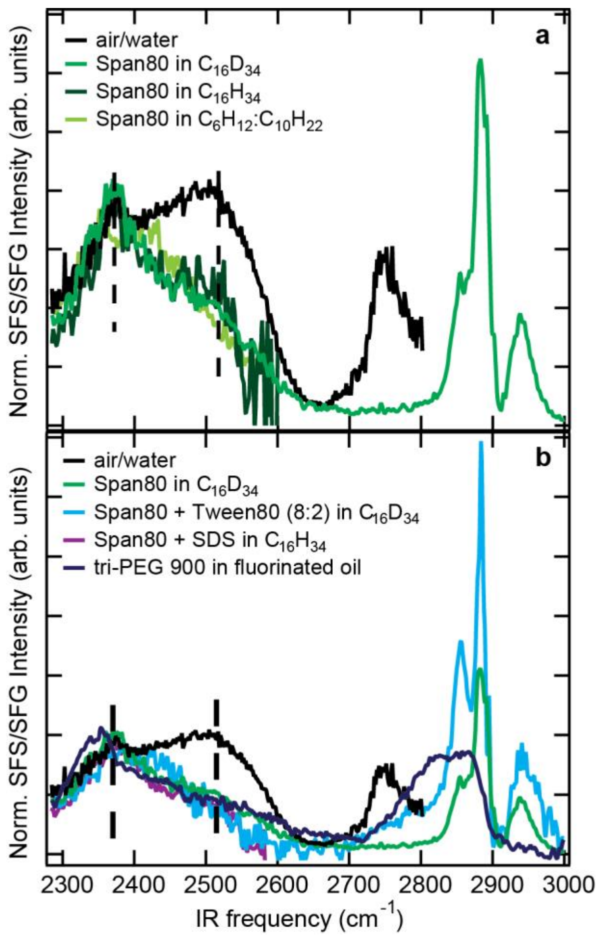

Supplementary Figure 2. Additional SFS data. SFS data for water droplets in different solvents (a): D_2O droplets in hydrogenated hexadecane (dark green) and deuterated hexadecane (green) and D_2O droplets in a mixture of cyclohexane and decane (light green). The concentration of D_2O was 1% and emulsions were stabilized with 5 mM Span80. The spectra are very similar to the one reported in Fig. 1c. SFS data for water droplets stabilized with different surfactants in different oils (b): 5 mM Span80 mixture in deuterated hexadecane (green), a 5 mM mixture of 84:16 weight fraction Span80:Tween80 in deuterated hexadecane (cyan), and a mixture of 10 mM SDS with 5 mM Span80 (purple). The addition of both anionic (SDS) and hydrophilic non-ionic surfactants (Tween80) does not result in significant changes of the SFS spectrum. Changing both the oil and the surfactant, using D_2O droplets prepared in HFE7500 fluorinated oil stabilized with a tri-block PEG-900 surfactant (dark blue) also yields a similar spectrum. Aqueous micron-sized drops dispersed in a perfluorinated oil, HFE-7500 (3M, USA), containing 1 wt% of a triblock co-polymer surfactant were produced according to the protocol in Ref. 1. Air/water planar SFG spectrum is shown in black.

Supplementary Note 1. Analysis of Span80 surface structure

In order to estimate the surface structure of the Span80 molecules we fitted the C-H mode region of the Span80 spectra and derived the molecular orientation from the tilt angle of the single methyl group present in the molecule. Supplementary Fig. 3a displays the SF spectrum of Span80 at the water droplet / d_{34}-hexadecane oil interface using the same polarization combination as used in the main text. In the C-H spectral region, the scattered SF intensity of the C-H modes is described by the following Lorentzian line shape expression[2]:

$$S_{\mathrm{SFS}}(\theta,\omega) \propto \left| A_{\mathrm{NR}}(\theta) f(\omega) e^{i\varphi_{\mathrm{NR}}} + \sum_i \frac{A_i(\theta)\gamma_i}{\omega-\omega_i+i\gamma_i} \right|^2, \qquad (1)$$

where $A_{\mathrm{NR}}(\theta)$ is the amplitude and $f(\omega)$ is the spectral shape of a weakly dispersive ('non-resonant') background, φ_{NR} is the phase of the background signal relative to that of the resonant signal, $A_i(\theta)$ is the amplitude of the ith vibrational mode with the resonance frequency ω_i and linewidth γ_i. The strength of the vibrational mode is proportional to A_i ($\omega = \omega_i$). The SFS spectra ($I_{\mathrm{SFS}}/I_{\mathrm{IR}}\mu_{\mathrm{FTIR}}$) were fit using Eq. 1, employing IGOR Pro 6 (WaveMetrics) and using Levenberg—Marquardt iterations in the fitting. The obtained fit is shown as the black line in Supplementary Fig. 3a and the fit parameters are given in Supplementary Table 1.

To determine the average tilt angle of the CH_3 group with respect to the droplet surface normal we used the r^-/r^+ ratio amplitude ratio. The details of this analysis as well as the used input values for the oil/water interface are described in the SI of Ref. 3. The r^-/r^+ scattered amplitude ratio was calculated using a hyperpolarizability ratio of $\beta^{(2)}_{aac}/\beta^{(2)}_{ccc}$ = 1.8. The result is plotted as a function of the tilt angle in Supplementary Fig. 3b. The experimental amplitude ratio obtained from the fit to the data (blue) in Supplementary Fig. 3a is 0.27, and indicated in Supplementary Fig. 3b as a rectangle (which includes the error bar). This value translates to a methyl group tilt angle of 10°±10°, i.e. the CH_3 group is oriented approximately parallel to the surface normal. Since there are no vibrational modes present in the SFS spectrum above 3000 cm^{-1}, the unsaturated =C-H mode is likely adopting an orientation that is nearly parallel to the interfacial plane. The structure is sketched in the inset of Supplementary Fig. 3a and results in a surface that is partially covered with CD groups (from the oil phase), CH modes from the Span80, and 6 (3) groups that can act as H-bond acceptors (donors) per Span80 molecule. Given the derived methyl group orientation, the H-bond acceptors and donors are likely pointing towards the water phase providing enough groups for the unbound OD water groups to H-bond with. Such a structure results in an absence of the free OD mode at 2745 cm^{-1} in Figs. 1 and 2.

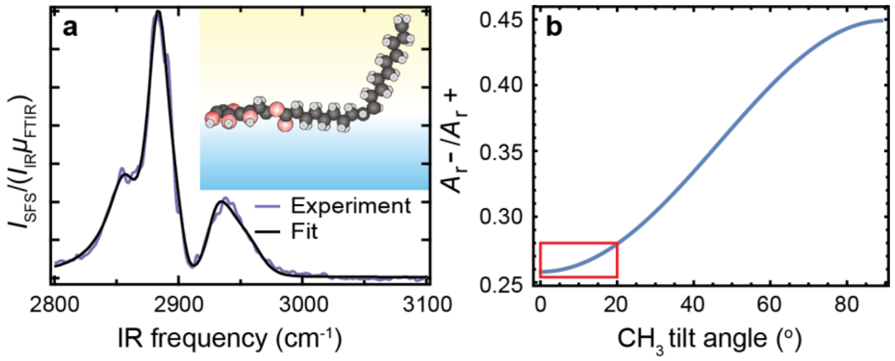

Supplementary Figure 3. Analysis of Span80 surface structure. a. Experimental (blue) and fitted (black) spectra in the CH region of water droplets stabilized with Span80 in d_{34}-hexadecane. The inset shows the orientation of the Span80 molecule at the oil/water interface as discussed in the text. b: dependence of A_{r-}/ A_{r+} ratio on the tilt angle.

mode	ω_i (cm^{-1})	Υ_i (cm^{-1})	A_i
r^+	2883	11	1
d+	2857	14	0.42
d^+_{FR}	2928	14	0.45
r^-	2965	20	-0.26
d^-	2905	11	-0.25

Supplementary Table 1. Fitted resonance frequency, amplitude and linewidth of the SFS spectrum in the CH region of water droplets stabilized with Span80 in d_{34}-hexadecane.

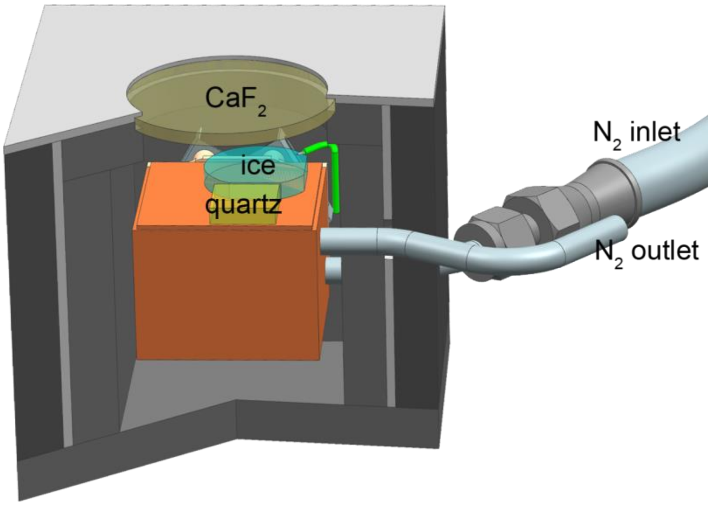

Supplementary Figure 4. Illustration of the temperature cell used for the experiments on basal ice. The temperature cell is cooled with liquid N_2. The temperature is monitored by a thermocouple welded into the ice and set by a heating foil resistance placed between the copper block and a copper plate. For reference purposes, a z-cut quartz crystal is placed adjacent to the ice.

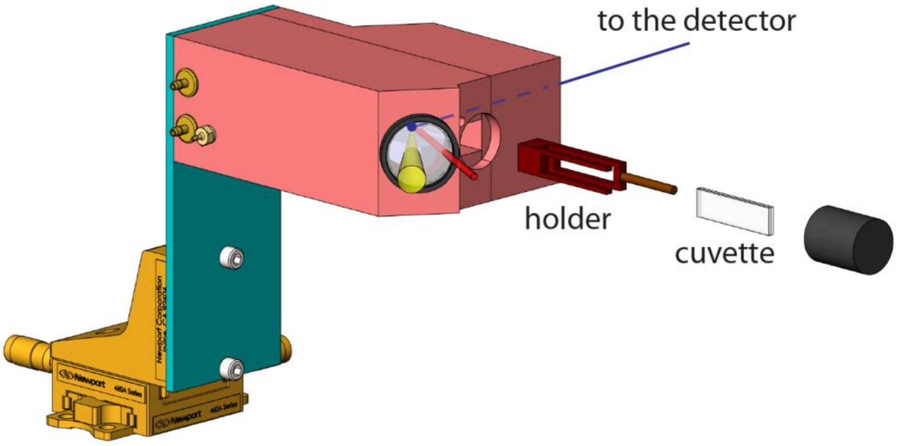

Supplementary Figure 5. Illustration of the temperature cell used in the SFS experiments. The yellow beam indicates the IR pulse, the red beam indicates the VIS pulse and the dashed blue line indicates the scattered SF light.

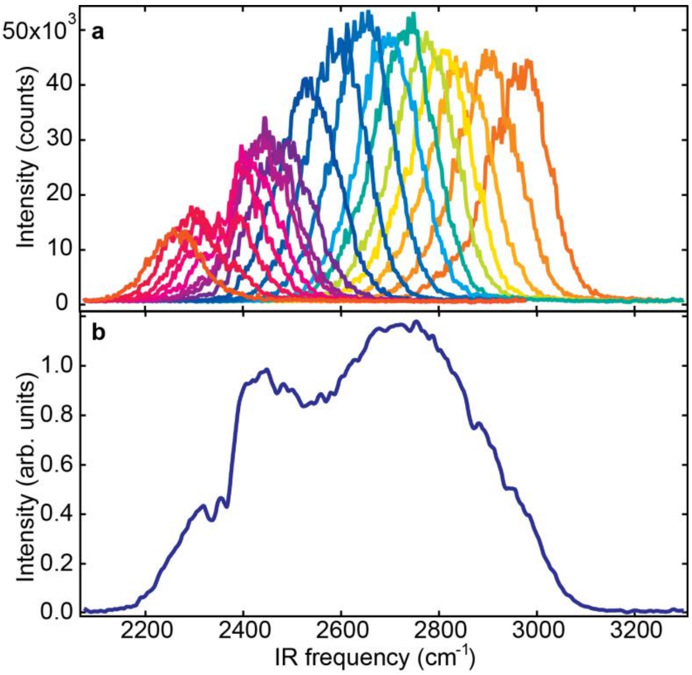

Supplementary Figure 6. IR spectra. a: SFG reflection mode spectra from a z-cut quartz crystal reflecting the spectral shape of the used IR pulses. b: The total IR spectrum resulting from the scan in panel a.

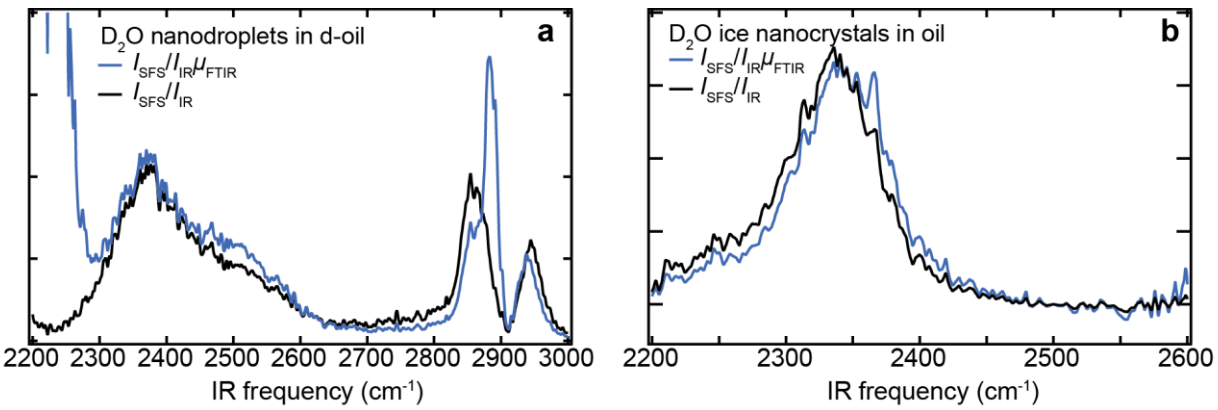

Supplementary Figure 7. Normalization procedure. The SFS spectra of Fig. 1c and 2b with (blue) and without (black) correction with the FTIR spectrum.

Parameter	Value
Opening angle between IR/VIS beams	15°
Scattering angle in the oil phase	45°
Number of expansion terms in the numerical algorithm	10
Effective orientation angle of the water molecules as represented by a δ distribution	39.2°
Non-zero independent $\beta^{(2)}$ components and their relative values[4,5]	β_{ccc}=1<br>β_{aac}=0.92
Particle diameter	200 nm
Polarization combination (SF,VIS,IR)	SSP

Supplementary Table 2. Parameters used for the nonlinear Mie calculation to correct the SFS spectra of D_2O droplets in oil for changes in the amplitude of the electromagnetic fields as they cross the droplet interface.

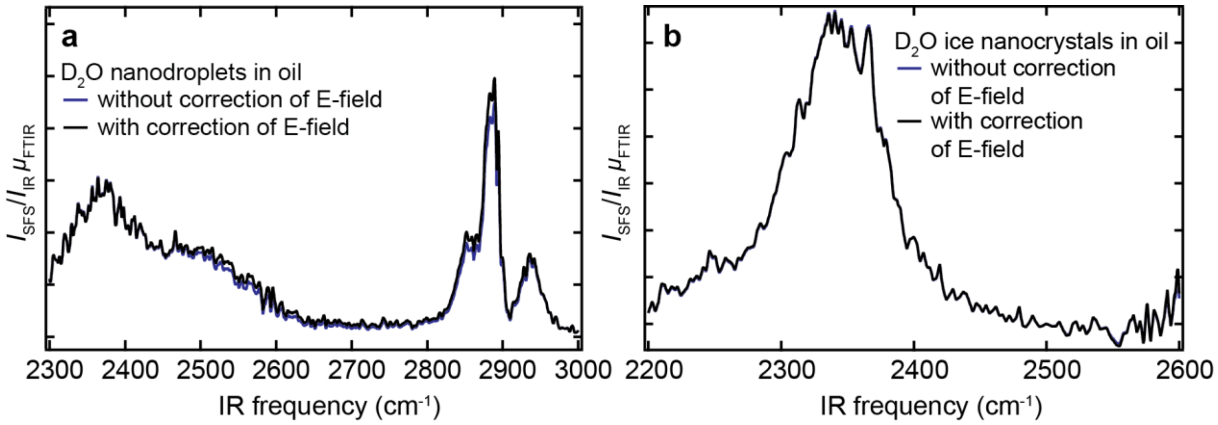

Supplementary Figure 8. Discontinuity in the interfacial electric fields. Comparison of normalized SFS spectra ($I_{\mathrm{SFS}}/I_{\mathrm{IR}}\mu_{FTIR}$) with (black) and without (blue) nonlinear Mie correction for D_2O droplets in d_{34}-hexadecane at room temperature (a) and D_2O ice nanocrystals in a decane:cyclohexane mixture (b).

Supplementary references

1 Etienne, G., Kessler, M. & Amstad, E. Influence of fluorinated surfactant composition on the stability of emulsion drops. *Macromol. Chem. Phys.* **218**, 1600365 (2017).

2 de Aguiar, H. B., Strader, M. L., de Beer, A. G. F. & Roke, S. Surface structure of SDS Surfactant and oil at the oil-in-water droplet liquid/liquid interface: A manifestation of a non-equilibrium surface state. *J. Phys. Chem. B* **115**, 2970-2978 (2011).

3 Chen, Y., Jena, K. C. & Roke, S. From Hydrophobic to Hydrophilic: The Structure and Density of the Hexadecane Droplet/Alkanol/Water Interface. *J. Phys. Chem. C* **119**, 17725-17734 (2015).

4 Zhang, W.-k. *et al.* Reconsideration of second-harmonic generation from isotropic liquid interface: Broken Kleinman symmetry of neat air/water interface from dipolar contribution. *J. Chem. Phys.* **123**, 224713 (2005).

5 de Beer, A. G. F. & Roke, S. Obtaining molecular orientation from second harmonic and sum frequency scattering experiments in water: Angular distribution and polarization dependence. *J. Chem. Phys.* **132**, 234702 (2010).
